# Supplementary material for: Complex‐centric proteome profiling by SEC‐SWATH‐MS
Source: Mol Syst Biol. 2019 Jan 14;15(1):e8438. doi: 10.15252/msb.20188438 (PMC6346213; doi:10.15252/msb.20188438)
Supplement: Supplementary file 6 — Dataset EV5 [file MSB-15-e8438-s006.zip › feature_plots_corum/778.pdf]

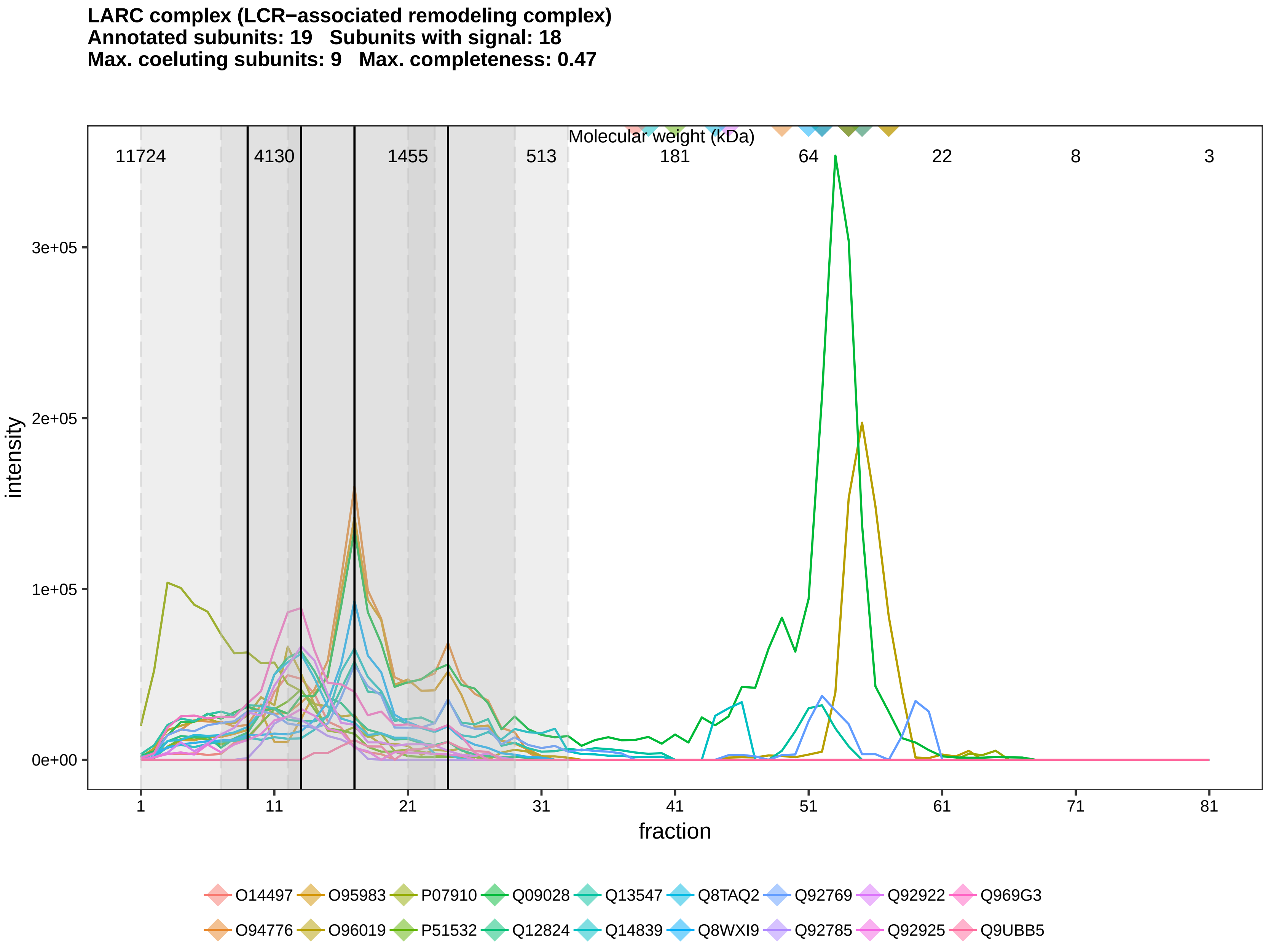
LARC complex (LCR−associated remodeling complex)
Annotated subunits: 19 Subunits with signal: 18
Max. coeluting subunits: 9 Max. completeness: 0.47
Molecular weight (kDa)
11724
4130
1455
513
181
64
22
8
3
intensity
3e+05
2e+05
1e+05
0e+00
fraction
1
11
21
31
41
51
61
71
81
O14497
O95983
P07910
Q09028
Q13547
Q8TAQ2
Q92769
Q92922
Q969G3
O94776
O96019
P51532
Q12824
Q14839
Q8WXI9
Q92785
Q92925
Q9UBB5
